# *CACNA1C* risk variant affects reward responsiveness in healthy individuals

**DOI:** 10.1038/tp.2014.100

**Published:** 2014-10-07

**Authors:** T M Lancaster, E A Heerey, K Mantripragada, D E J Linden

**Affiliations:** 1 Neuroscienceand Mental Health Research Institute and MRC Centre for Neuropsychiatric Genetics and Genomics, Cardiff University, Cardiff, UK; 2 School of Psychology, Cardiff University, Cardiff, UK; 3 School of Psychology, Bangor University, Bangor, UK

## Abstract

The variant at rs1006737 in the L-type voltage-gated calcium channel (alpha 1c subunit) *CACNA1C* gene is reliably associated with both bipolar disorder and schizophrenia. We investigated whether this risk variant affects reward responsiveness because reward processing is one of the central cognitive-motivational domains implicated in both disorders. In a sample of 164 young, healthy individuals, we show a dose-dependent response, where the rs1006737 risk genotype was associated with blunted reward responsiveness, whereas discriminability did not significantly differ between genotype groups. This finding suggests that the *CACNA1C* risk locus may have a role in neural pathways that facilitate value representation for rewarding stimuli. Impaired reward processing may be a transdiagnostic phenotype of variation in *CACNA1C* that could contribute to anhedonia and other clinical features common to both affective and psychotic disorders.

## Introduction

Although the heritability of psychiatric illness is high, the genetic mechanisms that confer susceptibility remain relatively unknown.^1,2^ Recent genome-wide association studies (GWAS) have repeatedly identified a risk variant in the *CACNA1C* gene, which encodes an L-type voltage-gated calcium channel (alpha 1C subunit). The risk variant (rs1006737: A allele) is significantly associated with bipolar disorder^3,4^ and schizophrenia,^5, 6, 7, 8^ supporting the hypothesis that *CACNA1C* contributes to the genetic overlap between these psychiatric disorders.

The rs1006737 risk variant has also been associated with several intermediate phenotypes such as neural activity during episodic^9, 10, 11, 12^ and working memory,^13^ emotional regulation,^14, 15, 16^ and verbal fluency.^17,18^ Numerous studies also suggest the rs1006737 variant may affect components of cognition, such as logical/working memory^19,20^ and clinical symptomology.^21,22^ Furthermore, emerging evidence supports a role for rs1006737 in the neural processing of reward^23^ and learning.^24^ Considering that the *CACNA1C* variant appears to transcend diagnostic boundaries, we hypothesize that the risk variant may affect intermediate phenotypes that are associated with several psychiatric illnesses.

A growing body of research suggests that the neural circuitry supporting reward processing may be a suitable platform of study, as impaired reward function is repeatedly observed as a component of neuropsychiatric disorders.^25, 26, 27, 28, 29, 30^ One promising candidate intermediate phenotype for several neuropsychiatric disorders is response bias, as measured by the probabilistic reward-learning task.^31^ The paradigm measures the development of response bias on the basis of a differential reinforcement schedule and indicates an individual's propensity to respond to reward. Reward responsiveness initially predicted an anhedonic phenotype,^31^ but has since been shown to be diminished in patients with bipolar disorder^32^ and more general depressive phenotypes.^33, 34, 35^

Reward responsiveness may also be modulated by dopaminergic innervation^36,37^ and interactions between stress and genes that influence the stress-axis.^38, 39, 40^ Additional evidence supports a role for gene variants that influence dopaminergic regulation.^41,42^ These associations are supported by findings that reward responsiveness during the task is moderately heritable.^43^ However, no studies have looked at the potential association between GWAS-identified risk variants and reward responsiveness. We consider *CACNA1C* rs1006737 as a candidate variant to probe for effects in reward responsiveness considering previous literature suggests (a) *CACNA1C* is associated putative effects on reward/learning;^23,24^ (b) *CACNA1C* is associated with mood disorders at genome-wide level^3,4,7^ and (c) reward responsiveness is diminished as a component of these psychiatric illnesses.^32, 33, 34, 35^ As the A allele at rs1006737 is overrepresented in neuropsychiatric illness, we anticipate that the genotype group with the A risk allele will show blunted reward responsiveness compared with individuals who do not carry a copy of the risk allele.

## Materials and methods

### Participants

Bangor site: 131 subjects were recruited from Bangor University and genotyped for the *CACNA1C* variant (rs1006737). Participants in this panel were recruited by advertisement, from among the University community (for example, students, employees) on the basis of the following self-reported criteria: western European descent; no experience of psychiatric/neurological symptoms or diagnoses in either themselves or first-degree relatives; no illegal (or recreational) substance use/dependence (excluding nicotine) and no alcohol abuse/dependence. Cardiff site: 34 Caucasian volunteers were genotyped for the *CACNA1C* variant (rs1006737). No participants reported any current mental illness^44^ or use of psychotropic medication. The study was approved by the ethics committee of each University and written, informed consent was given by all participants before gDNA extraction and participation. A total of 164 healthy individuals were included in the study (sample demographics are described in Table 1). *CACNA1C* rs1006737 genotype frequencies did not significantly differ between sites (*χ*^2^=1.488, *P*=0.475; Table 1). Genotype frequencies for rs1006737 did not deviate from Hardy–Weinberg equilibrium (*χ*^2^=1.51, *P*=0.217; Table 1). On the basis of the effect size from a previous candidate gene-response bias study with a similar sample size,^38^ we anticipate to see a small effect size (Cohen's *d*=0.38). A power calculation^45^ suggested that we had 78% power to detect an effect of this size for rs1006737 on response bias (*α*=0.05, one-sided).

### rs1006737 SNP genotyping

Bangor site: genomic DNA was obtained from saliva using Oragene OG-500 saliva kits for 136 participants. Genotyping of rs1006737 was performed using the Illumina Golden Gate assay (Illumina, San Diego, CA, USA) using the BeadXpress platform, which allows high-throughput multiplex genotyping of SNPs. Assays were designed for the experiment using Illumina's Assay Design Tool (http://support.illumina.com/array/array_software/assay_design_tool.ilmn). SNP sequence: 5′-ACTTGGCTCTATCAAAGTCTTGCTATCAATTACATAAGTTCCATTCCATCTCAGCCCGAA[A/G]TGTTTTCAGAGCCGGAGACCTCACAGTGTCTCTCAGGACAGTACCTTTCAGGTTTGAATG-3′. Nucleic acid concentration was evaluated using PicoGreen assay (Life Technologies, Carlsbad, CA, USA). Golden Gate genotyping was performed according to manufacturer's protocols. Genotype calling and annotation were performed using GenomeStudio (Illumina). *CACNA1C* rs1006737 was not available for five individuals from the Bangor site.

Cardiff site: genomic DNA was obtained from saliva using Oragene OG-500 saliva kits for 37 participants. *CACNA1C* rs1006737 was genotyped using custom SNP genotyping arrays from Illumina (Illumina). Individuals were excluded for ambiguous sex, cryptic relatedness up to third-degree relatives by identity of descent, genotyping completeness <97%, and non-European ethnicity admixture detected as outliers in iterative EIGENSTRAT analyses of an LD-pruned data set.^46^ Thirty four of the 37 individuals included in the sample had genotype data available for rs1006737.

### Experimental Procedure

To measure reward responsiveness, we used a line discrimination task with asymmetric reinforcement, closely modelled after that described in Pizzagalli *et al.*^31^ and Heerey *et al.*^47^ Asymmetric reinforcement, in which correct responses to one stimulus receive more frequent rewards than correct responses to another, leads to the development of response bias by increasing participants' likelihood of reporting the more frequently reinforced stimulus.^48^ It is hypothesized that individuals who develop greater levels of response bias are more responsive to rewards.^31^ Trials began with a fixation cross (500 ms), followed by the presentation of a cartoon face with no mouth. After 500 ms, either a short (22 mm) or long (24 mm) mouth appeared on the face. It was visible for 100 ms before disappearing. The face remained on screen until the participant responded with a button press indicating the presence of either the short or long mouth. Following the response, participants saw a screen that either displayed feedback (‘correct +5 pence') or remained blank (no-feedback trials) for 1750 ms. Participants completed three blocks of 100 trials. Both versions of the mouth appeared equally often in pseudo-random order such that there were no more than four successive trials of the same mouth. Participants received reward feedback on 40 correct responses per block. To induce a reward-related response bias in the task, we distributed the rewards asymmetrically across the mouths. The more frequently reinforced mouth received 30 rewards per block and the remaining 10 rewards occurred after responses to the other mouth. We used a pseudo-random reward schedule such that no more than three correct trials in a row received reinforcements. Participants never received feedback on incorrect trials. When positive feedback was scheduled for a trial and the participant answered incorrectly, the reinforcement was postponed until a later unreinforced, correct trial (of the same mouth length) occurred. The length (short or long) of the more frequently reinforced mouth was counterbalanced across participants. All trials where reaction times faster than 200 ms and slower than 3000 ms were removed from the analysis as previously described.^47^ We measured the frequency of each participant's reward feedback schedule on the basis of the number of positive rewards (‘correct +5 pence') they received. The maximum bonus was £6 and individuals who earned <£5 were excluded from the analysis (n=1). The reward feedback schedule received did not significantly differ between gender (F_1,163_=1.580, *P*=0.210); rs1006737 genotype group (F_2,163_=0.180, *P*=0.836); across sample site (F_1,163_=0.525, *P*=0.47) and did not correlate with age (*r*=0.084, *P*=0.285).

A post-task debriefing interview confirmed that no participants were aware of the reinforcement asymmetry. We used a standard signal detection analysis to calculate *d*', a measure of discrimination accuracy [*d*=*z*(H)−*z*(F)] and ‘criterion,' the degree to which participants showed a bias towards the more frequently reinforced mouth (*c*=−1/2[*z*(H)=*z*(F)]^47,48^). Please note that we reversed the values for criterion in the analysis (positive values represent a higher propensity for developing response bias), for ease of interpretation.

## Results

### Effects of demographic factors

There were no site specific differences in participants' ability to discriminate between the mouths (F_1,163_=1.999, *P*=0.159) or in the degree to which they developed response biases (F_1,164_=1.095, *P*=0.297). Therefore, data from the two sites were combined. Moreover, data from men and women were analysed together, as there were no significant differences between gender groups in either discriminability (*d*') or criterion across the whole group (both *P*-values >0.354) or within *CACNA1C* rs1006737 genotype groups (all *P*-values >0.111). There was a significant positive association between age and discriminability at block 1 (*r*=0.155, *P*=0.048). Therefore age was entered as a covariate into a mixed-model analysis of covariance to assess the potential effects of *CACNA1C* rs1006737 on discriminability (*d*'). There were no associations between age and criterion within genotype groups (all *P-*values >0.306). One additional individual's discrimination ability was above the expected frequency (GG rs1006737 genotype); however, removing this individual did not significantly affect any demographic or genetic analysis. There were no significant outliers for the criterion measure.^49^ We therefore used mixed-model analyses of variance to compare criterion across task blocks (1–3) and genotype groups (AA=23; AG=62; GG=79). We also ran the same mixed-model analyses of variance for criterion using a dominant genetic model (AA/AG=85; GG=79) as previously described.^9,23,24^

### *CACNA1C* genotype effects

**Figure 1:**
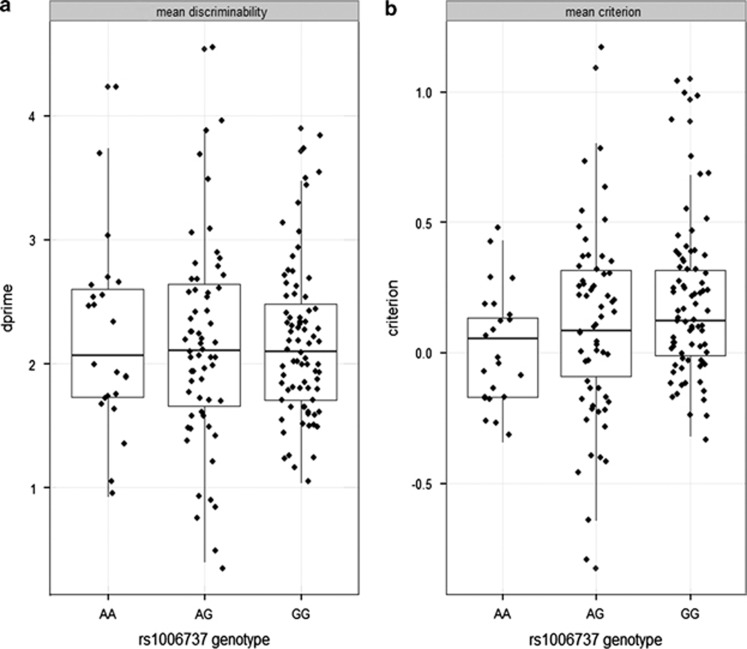
(**a**) Mean discriminability of long/short mouth types (*d*') between the *CACNA1C* rs1006737 genotype groups. (**b**) Differences in mean criterion (response bias) between the *CACNA1C* rs1006737 genotype groups. Horizontal lines represent median and represent 25th and 75th percentile. Note: positive values for criterion reflect an increase in response bias.

**Figure 2:**
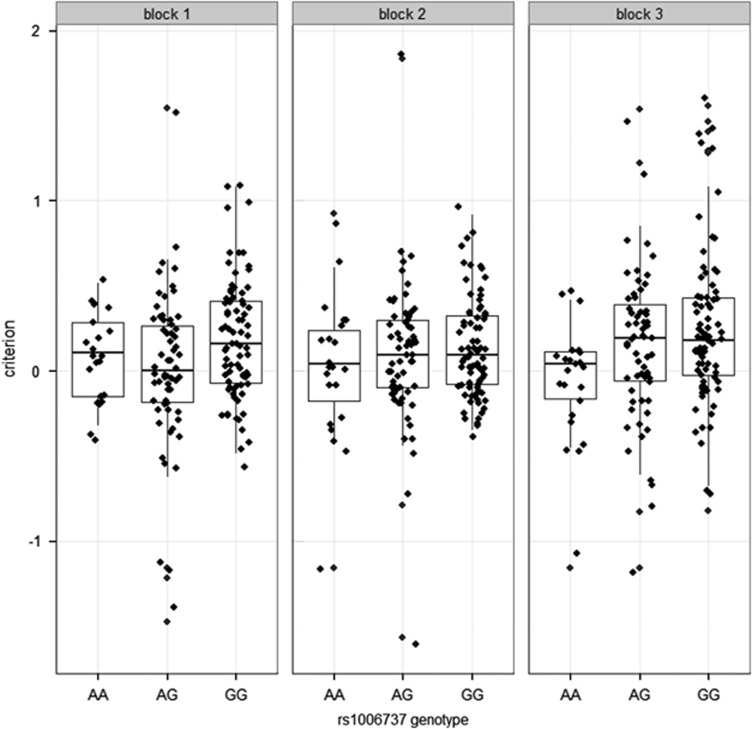
Data represent the criterion (response bias) for each of the three *CACNA1C* rs1006737 genotype groups (as Figure 1b), but split across the three experimental trial blocks. Horizontal lines represent median and represent 25th and 75th percentile. Note: positive values for criterion reflect an increase in response bias.

Participants performed the line discrimination task equally well, regardless of genotype group (additive genetic model: F_2,160_=0.006, *P*=0.994; dominant genetic model; F_1,162_=0.000, *P*=0.995; see Figure 1a). However, we observed *CACNA1C* genotype dependent differences in reward responsiveness, such that the risk allele was associated with diminished reward responsiveness (additive genetic model: F_2,162_=3.374, *P*=0.037, n_p_^2^=0.040; dominant model: F_1,163_=5.656, *P*=0.019, n_p_^2^=0.034; see Figure 1b). Furthermore, in the additive genetic model, there was a significant task block × genotype interaction (F_2,161_=2.417, *P*=0.05, n_p_^2^=0.029), suggesting that genotype differences in reward responsiveness were larger toward the end of the task (see Figure 2). One-way analysis of variance confirmed that the largest between group difference occurred in task block 3 (F_2,163_=3.810, *P*=0.024, n_p_^2^=0.045). *Post hoc* independent-sample *t*-tests also revealed a dose-dependent response, in which the largest differences in mean response bias occurred between the AA and GG groups (pairwise comparison; p_corrected_=0.011; Cohen's *d*= 0.66). Independent samples *t*-test using the dominant genetic model suggested that A risk allele carriers (AA/AG) also had pronounced deficits in reward responsiveness compared with the nonrisk (GG) genotype group (*P*=0.019; Cohen's *d*=0.37). On the basis of our smallest effect size (Cohen's *d*=0.37), we estimate we had 76% power to detect a significant main effect of rs1006737 on mean criterion (one-sided; *α*=0.05).

### *CACNA1C* genotype X task block effects

To explore the block × rs1006737 genotype interaction, we explored variations in response bias for each genotype group at each level. We calculated the change in criterion over the task (Δresponse bias=block 3−block 1). A one-way analysis of variance suggested that rs1006737 genotype related to an individual's propensity to develop response bias (additive mode; F_2,163_=2.950, *P*=0.05, which was driven by a difference between the AA and AG genotype *p*_corrected_=0.05). We additionally performed *post hoc* one-sample *t*-tests to test whether the rs1006737 genotype groups (AA/AG/GG) response bias significantly differed from 0 at each block (1–3). The AA homozygous risk group did not begin with or develop any response bias at any block (*P*>0.2 in all cases). The AG heterozygous risk group showed a trend towards developing response bias (block 1: NS; block 2: *t*=1.807, *P*=0.076; block 3: *t*=2.643, *P*=0.010), but these results were not significant after correction for multiple comparisons. However, the GG homozygous group showed significant response bias during all three blocks (block 1: *t*=4.446, *P*<0.001; block 2: *t*=3.996, *P*<0.001 and block 3: *t*=4.675, *P*<0.001). This analysis indicates that the GG genotype showed response bias early in the task, the AG genotype group started to show a Δresponse bias in later trials, but in comparison, the AA risk group showed no evidence of developing response bias at any point throughout the paradigm (see Figure 2).

## Discussion

Previous research suggests that the *CACNA1C* rs1006737 risk variant is associated with heritable neuropsychiatric disorders.^3,4,8,50^ Reward responsiveness is potentially heritable^43^ and has been shown to be disrupted in patients with bipolar and unipolar depression.^32, 33, 34, 35^ Response bias is also blunted in remitted patients, suggesting that reward responsiveness may serve as trait marker.^33^ We demonstrate for the first time that the *CACNA1C* risk variant (rs1006737: A allele) modulates an individual's propensity to respond to reward, without disrupting general task discrimination ability. It is an attractive feature of the reward-responsiveness paradigm that it allows for the investigation of cognitive biases in situations of normal discriminability, which makes it a particularly sensitive trait marker for healthy at-risk populations.

This finding adds to a growing body of literature suggesting that the *CACNA1C* variant may affect the neural mechanisms underlying reward processing and learning.^23,24,51^ Blunted reward responsiveness was seen across the whole task for AA genotype, the rs1006736 AG genotype group showed response bias towards the end of the task, whereas the GG nonrisk group showed reward responsiveness throughout the task. We suggest that rs1006737 ‘A' risk allele dose may contribute to psychopathology by affecting the rate of reinforcement–based learning, although this would need to be formally tested in extended versions of the paradigm. This finding is comparable to previous reports where impairment in learning was most pronounced in rs1006737 A carriers at the beginning of the task.^24^ These results offer novel insight into how *CACNA1C* may confer susceptibility to the neuropsychiatric illness characterized by reduced reward responsiveness and impairments in reward-based learning. The association between *CACNA1C* rs1006737 A allele and blunted response bias may point to a genetic basis for anhedonia, which is a symptom common to affective and psychotic disorders.^27,32,52, 53, 54^ Although evidence suggests that anhedonia is a residual trait underlying euthymic bipolar disorder,^54^ it is less straightforward to align our findings with bipolar phenotypes associated with the hyperthymic state, as it is speculated that manic states could be associated with a potentiated rather than blunted response bias.^32^

Although the genetic architecture of reward-related deficits and related clinical symptoms (such as anhedonia) remains unknown, reward responsiveness may be a promising neurobiological process that links novel risk loci (such as SNPs identified via genome-wide association studies) with core clinical symptoms. It could also emerge as a promising surrogate marker for treatment effects. A limitation of the present study was that we did not screen for nicotine use, which may have an interactive effect on reward responsiveness.^55^ We suggest that the effect of the rs1006737 (and any other common variant) on reward responsiveness are likely to be small^51^ and thus, our sample may have been underpowered. We therefore recommend that our results should be treated with caution until replicated in larger, independent samples.^56, 57, 58^ Additional studies could further help to verify how much variance in response bias associated with rs1006737 genotype. Furthermore, we cannot be sure whether rs1006737 is responsible for these effects or rather SNPs that are in linkage disequilibrium with the variant. Nevertheless, recent GWAS implicate *CACNA1C* as a suitable candidate for probing intermediate traits associated with multiple neuropsychiatric illness.^59^ It is possible that additive variations within CACNA1C^12^ or interactions with other genes^60^ could further modulate reward responsiveness and explain larger proportions of variance.

In conclusion, our results suggest that the genome-wide identified psychiatric risk locus on *CACNA1C* (rs1006737) may affect an individual's ability to respond to reward. An attractive feature of the rs1006737 ‘A' risk allele is its association with increased *CACNA1C* mRNA expression in cortical tissue,^16^ which offers putative mechanistic insight into how the variant may affect neural circuitry. A growing body of knowledge implicates the *CACNA1C* gene product in animal models of reward processing,^61, 62, 63^ therefore translational animal models homologous to reward responsiveness^64^ may also give further insight into the molecular mechanisms underlying the association between *CACNA1C* and reward responsiveness. Future studies could also explore the neural dynamics that support reward responsiveness to understand how *CACNA1C* may exert these effects.

## Figures and Tables

**Table 1:** Descriptive statistics for *CACNA1C* rs1006737

CACNA1C *rs1006737*	*AA*	*AG*	*GG*	P
*Bangor site*				
Genotype frequency^a^	*N*=16	*N*=50	*N*=64	0.475
Age^b^	22.06 (±3.907)	21.72 (±4.257)	21.61 (±3.736)	0.914
Gender (M/F)^c^	*N*=8/*N*=8	*N*=27/*N*=23	*N*=22/*N*=42	0.097
				
*Cardiff site*				
Genotype frequency^a^	*N*=7	*N*=12	*N*=15	0.475
Age^b^	24.14 (±2.911)	24.50 (±4.056)	25.13 (±7.100)	0.919
Gender (M/F)^c^	*N*=2/*N*=5	*N*=6/*N*=6	*N*=7/*N*=8	0.639
				
*Combined sample*				
Genotype frequency^d^	*N*=23	*N*=62	*N*=79	0.217
Age^b^	22.70 (±3.698)	22.21 (±4.315)	22.23 (±4.717)	0.899
Gender (M/F)^c^	*N*=10/*N*=13	*N*=33/*N*=30	*N*=29/*N*=50	0.174

a *CACNA1C* rs1006737 genotype frequencies between sample site tested with *χ*^2^.

b Group differences tested with one-way analysis of variance.

c Gender group differences tested with *χ*^2^.

d Group differences tested for Hardy–Weinberg equilibrium. Table shows means (except where noted; s.d. appear in parentheses).

## References

[bib1] International Schizophrenia Consortium Purcell SM Wray NR Stone JL Visscher PM O'Donovan MC Common polygenic variation contributes to risk of schizophrenia and bipolar disorder Nature 2009 460 748 752 19571811 10.1038/nature08185 PMC3912837

[bib2] Muglia P Tozzi F Galwey NW Francks C Upmanyu R Kong XQ Genome-wide association study of recurrent major depressive disorder in two European case-control cohorts Mol Psychiatry 2010 15 589 601 19107115 10.1038/mp.2008.131

[bib3] Ferreira MA O'Donovan MC Meng YA Jones IR Ruderfer DM Jones L Collaborative genome-wide association analysis supports a role for ANK3 and CACNA1C in bipolar disorder Nat Genet 2008 40 1056 1058 18711365 10.1038/ng.209 PMC2703780

[bib4] Green EK Hamshere M Forty L Gordon-Smith K Fraser C Russell E Replication of bipolar disorder susceptibility alleles and identification of two novel genome-wide significant associations in a new bipolar disorder case-control sample Mol Psychiatry 2013 18 1302 1307 23070075 10.1038/mp.2012.142 PMC3971368

[bib5] Lett TA Zai CC Tiwari AK Shaikh SA Likhodi O Kennedy JL ANK3, CACNA1C and ZNF804A gene variants in bipolar disorders and psychosis subphenotype World J Biol Psychiatry 2011 12 392 397 21767209 10.3109/15622975.2011.564655

[bib6] Nyegaard M Demontis D Foldager L Hedemand A Flint TJ Sørensen KM CACNA1C (rs1006737) is associated with schizophrenia Mol Psychiatry 2010 15 119 121 20098439 10.1038/mp.2009.69

[bib7] Green EK Grozeva D Jones I Jones L Kirov G Caesar S The bipolar disorder risk allele at CACNA1C also confers risk of recurrent major depression and of schizophrenia Mol Psychiatry 2010 15 1016 1022 19621016 10.1038/mp.2009.49 PMC3011210

[bib8] Casamassima F Huang J Fava M Sachs GS Smoller JW Cassano GB Phenotypic effects of a bipolar liability gene among individuals with major depressive disorder Am J Med Genet B Neuropsychiatr Genet 2010 153B 303 309 19388002 10.1002/ajmg.b.30962

[bib9] Krug A Witt SH Backes H Dietsche B Nieratschker V Shah NJ A genome-wide supported variant in CACNA1C influences hippocampal activation during episodic memory encoding and retrieval Eur Arch Psychiatry Clin Neurosci 2014 264 103 110 23860750 10.1007/s00406-013-0428-x

[bib10] Erk S Meyer-Lindenberg A Schmierer P Mohnke S Grimm O Garbusow M Hippocampal and frontolimbic function as intermediate phenotype for psychosis: evidence from healthy relatives and a common risk variant in CACNA1C Biol Psychiatry 2014 76 466 475 24411473 10.1016/j.biopsych.2013.11.025

[bib11] Erk S Meyer-Lindenberg A Schnell K Opitz von Boberfeld C Esslinger C Kirsch P Brain function in carriers of a genome-wide supported bipolar disorder variant Arch Gen Psychiatry 2010 67 803 811 20679588 10.1001/archgenpsychiatry.2010.94

[bib12] Erk S Meyer-Lindenberg A Linden D Lancaster T Mohnke S Grimm O Replication of brain function effects of a genome-wide supported psychiatric risk variant in the CACNA1C gene and new multi-locus effects Neuroimage 2014 94 147 154 24642287 10.1016/j.neuroimage.2014.03.007

[bib13] Paulus FM Bedenbender J Krach S Pyka M Krug A Sommer J Association of rs1006737 in CACNA1C with alterations in prefrontal activation and fronto-hippocampal connectivity Hum Brain Mapp 2014 35 1190 1200 23404764 10.1002/hbm.22244 PMC6869796

[bib14] Tesli M Skatun KC Ousdal OT Brown AA Thoresen C Agartz I CACNA1C risk variant and amygdala activity in bipolar disorder, schizophrenia and healthy controls PLoS One 2013 8 e56970 23437284 10.1371/journal.pone.0056970 PMC3577650

[bib15] Wang F McIntosh AM He Y Gelernter J Blumberg HP The association of genetic variation in CACNA1C with structure and function of a frontotemporal system Bipolar Disord 2011 13 696 700 22085483 10.1111/j.1399-5618.2011.00963.x PMC3233238

[bib16] Bigos KL Mattay VS Callicott JH Straub RE Vakkalanka R Kolachana B Genetic variation in CACNA1C affects brain circuitries related to mental illness Arch Gen Psychiatry 2010 67 939 945 20819988 10.1001/archgenpsychiatry.2010.96 PMC3282053

[bib17] Backes H Dietsche B Nagels A Konrad C Witt SH Rietschel M Genetic variation in CACNA1C affects neural processing in major depression J Psychiatr Res 2014 53 38 46 24612926 10.1016/j.jpsychires.2014.02.003

[bib18] Krug A Nieratschker V Markov V Krach S Jansen A Zerres K Effect of CACNA1C rs1006737 on neural correlates of verbal fluency in healthy individuals Neuroimage 2010 49 1831 1836 19781653 10.1016/j.neuroimage.2009.09.028

[bib19] Hori H Yamamoto N Fujii T Teraishi T Sasayama D Matsuo J Effects of the CACNA1C risk allele on neurocognition in patients with schizophrenia and healthy individuals Sci Rep 2012 2 634 22957138 10.1038/srep00634 PMC3434390

[bib20] Zhang Q Shen Q Xu Z Chen M Cheng L Zhai J The effects of CACNA1C gene polymorphism on spatial working memory in both healthy controls and patients with schizophrenia or bipolar disorder Neuropsychopharmacology 2012 37 677 684 22012475 10.1038/npp.2011.242 PMC3260980

[bib21] Strohmaier J Amelang M Hothorn LA Witt SH Nieratschker V Gerhard D The psychiatric vulnerability gene CACNA1C and its sex-specific relationship with personality traits, resilience factors and depressive symptoms in the general population Mol Psychiatry 2013 18 607 613 22665259 10.1038/mp.2012.53

[bib22] Roussos P Bitsios P Giakoumaki SG McClure MM Hazlett EA New AS CACNA1C as a risk factor for schizotypal personality disorder and schizotypy in healthy individuals Psychiatry Res 2013 206 122 123 22985546 10.1016/j.psychres.2012.08.039 PMC4176879

[bib23] Wessa M Linke J Witt SH Nieratschker V Esslinger C Kirsch P The CACNA1C risk variant for bipolar disorder influences limbic activity Mol Psychiatry 2010 15 1126 1127 20351721 10.1038/mp.2009.103

[bib24] Dietsche B Backes H Laneri D Weikert T Witt SH Rietschel M The impact of a CACNA1C gene polymorphism on learning and hippocampal formation in healthy individuals: a diffusion tensor imaging study Neuroimage 2014 89 256 261 24269271 10.1016/j.neuroimage.2013.11.030

[bib25] Bogdan R Pizzagalli DA Acute stress reduces reward responsiveness: implications for depression Biol Psychiatry 2006 60 1147 1154 16806107 10.1016/j.biopsych.2006.03.037 PMC2288705

[bib26] Der-Avakian A Markou A The neurobiology of anhedonia and other reward-related deficits Trends Neurosci 2012 35 68 77 22177980 10.1016/j.tins.2011.11.005 PMC3253139

[bib27] Huys QJ Pizzagalli DA Bogdan R Dayan P Mapping anhedonia onto reinforcement learning: a behavioural meta-analysis Biol Mood Anxiety Disord 2013 3 12 23782813 10.1186/2045-5380-3-12 PMC3701611

[bib28] Strauss GP Gold JM A new perspective on anhedonia in schizophrenia Am J Psychiatry 2012 169 364 373 22407079 10.1176/appi.ajp.2011.11030447 PMC3732829

[bib29] Hasler G Can the neuroeconomics revolution revolutionize psychiatry Neurosci Biobehav Rev 2012 36 64 78 21550365 10.1016/j.neubiorev.2011.04.011

[bib30] Sharp C Monterosso J Montague PR Neuroeconomics: a bridge for translational research Biol Psychiatry 2012 72 87 92 22727459 10.1016/j.biopsych.2012.02.029 PMC4096816

[bib31] Pizzagalli DA Jahn AL O'Shea JP Toward an objective characterization of an anhedonic phenotype: a signal-detection approach Biol Psychiatry 2005 57 319 327 15705346 10.1016/j.biopsych.2004.11.026 PMC2447922

[bib32] Pizzagalli DA Goetz E Ostacher M Iosifescu DV Perlis RH Euthymic patients with bipolar disorder show decreased reward learning in a probabilistic reward task Biol Psychiatry 2008 64 162 168 18242583 10.1016/j.biopsych.2007.12.001 PMC2464620

[bib33] Pechtel P Dutra SJ Goetz EL Pizzagalli DA Blunted reward responsiveness in remitted depression J Psychiatr Res 2013 47 1864 1869 24064208 10.1016/j.jpsychires.2013.08.011 PMC3978009

[bib34] Vrieze E Pizzagalli DA Demyttenaere K Hompes T Sienaert P de Boer P Reduced reward learning predicts outcome in major depressive disorder Biol Psychiatry 2013 73 639 645 23228328 10.1016/j.biopsych.2012.10.014 PMC3602158

[bib35] Pizzagalli DA Iosifescu D Hallett LA Ratner KG Fava M Reduced hedonic capacity in major depressive disorder: evidence from a probabilistic reward task J Psychiatr Res 2008 43 76 87 18433774 10.1016/j.jpsychires.2008.03.001 PMC2637997

[bib36] Pizzagalli DA Evins AE Schetter EC Frank MJ Pajtas PE Santesso DL Single dose of a dopamine agonist impairs reinforcement learning in humans: behavioral evidence from a laboratory-based measure of reward responsiveness Psychopharmacology 2008 196 221 232 17909750 10.1007/s00213-007-0957-y PMC2268635

[bib37] Santesso DL Evins AE Frank MJ Schetter EC Bogdan R Pizzagalli DA Single dose of a dopamine agonist impairs reinforcement learning in humans: evidence from event-related potentials and computational modeling of striatal-cortical function Hum Brain Mapp 2009 30 1963 1976 18726908 10.1002/hbm.20642 PMC3034238

[bib38] Bogdan R Perlis RH Fagerness J Pizzagalli DA The impact of mineralocorticoid receptor ISO/VAL genotype (rs5522) and stress on reward learning Genes Brain Behav 2010 9 658 667 20528958 10.1111/j.1601-183X.2010.00600.x PMC2921022

[bib39] Bogdan R Santesso DL Fagerness J Perlis RH Pizzagalli DA Corticotropin-releasing hormone receptor type 1 (CRHR1) genetic variation and stress interact to influence reward learning J Neurosci 2011 31 13246 13254 21917807 10.1523/JNEUROSCI.2661-11.2011 PMC3185047

[bib40] Nikolova Y Bogdan R Pizzagalli DA Perception of a naturalistic stressor interacts with 5-HTTLPR/rs25531 genotype and gender to impact reward responsiveness Neuropsychobiology 2012 65 45 54 22094432 10.1159/000329105 PMC3238029

[bib41] Goetz EL Hariri AR Pizzagalli DA Strauman TJ Genetic moderation of the association between regulatory focus and reward responsiveness: a proof-of-concept study Biol Mood Anxiety Disord 2013 3 3 23369671 10.1186/2045-5380-3-3 PMC3570330

[bib42] Lancaster TM Linden DE Heerey EA COMT val158met predicts reward responsiveness in humans Genes Brain Behav 2012 11 986 992 10.1111/j.1601-183X.2012.00838.x 22900954

[bib43] Bogdan R Pizzagalli DA The heritability of hedonic capacity and perceived stress: a twin study evaluation of candidate depressive phenotypes Psychol Med 2009 39 211 218 18507876 10.1017/S0033291708003619 PMC2628414

[bib44] Goldberg D Williams P A User's Guide to the General Health Questionnaire NFER-Nelson: Slough, UK1988.

[bib45] Champely S pwr: Basic functions for power analysis. R package version 1.1.1 2012Available at http://CRAN.R-project.org/package=pwr .

[bib46] Price AL Patterson NJ Plenge RM Weinblatt ME Shadick NA Reich D Principal components analysis corrects for stratification in genome-wide association studies Nat Genet 2006 38 904 909 16862161 10.1038/ng1847

[bib47] Heerey EA Bell-Warren KR Gold JM Decision-making impairments in the context of intact reward sensitivity in schizophrenia Biol Psychiatry 2008 64 62 69 18377874 10.1016/j.biopsych.2008.02.015 PMC2613513

[bib48] Macmillan NA Creelman CD Detection Theory: A User's Guide2 edn, Lawrence Erlbaum Associates: Mahwah/London, UK 2005

[bib49] Hoaglin DC Iglewicz B Fine-tuning some resistant rules for outlier labeling J Am Stat Assoc 1987 82 1147 1149

[bib50] Wray NR Pergadia ML Blackwood DH Penninx BW Gordon SD Nyholt DR Genome-wide association study of major depressive disorder: new results, meta-analysis, and lessons learned Mol Psychiatry 2012 17 36 48 21042317 10.1038/mp.2010.109 PMC3252611

[bib51] Bhat S Dao DT Terrillion CE Arad M Smith RJ Soldatov NM CACNA1C (Cav1.2) in the pathophysiology of psychiatric disease Prog Neurobiol 2012 99 1 14 22705413 10.1016/j.pneurobio.2012.06.001 PMC3459072

[bib52] Pizzagalli DA Depression, stress, and anhedonia: toward a synthesis and integrated model Annu Rev Clin Psychol 2014 10 393 423 24471371 10.1146/annurev-clinpsy-050212-185606 PMC3972338

[bib53] Pizzagalli DA The "anhedonia paradox" in schizophrenia: insights from affective neuroscience Biol Psychiatry 2010 67 899 901 20435208 10.1016/j.biopsych.2010.02.022 PMC2864781

[bib54] Di Nicola M De Risio L Battaglia C Camardese G Tedeschi D Mazza M Reduced hedonic capacity in euthymic bipolar subjects: a trait-like feature J Affect Disord 2013 147 446 450 23122985 10.1016/j.jad.2012.10.004

[bib55] Barr RS Pizzagalli DA Culhane MA Goff DC Evins AE A single dose of nicotine enhances reward responsiveness in nonsmokers: implications for development of dependence Biol Psychiatry 2008 63 1061 1065 17976537 10.1016/j.biopsych.2007.09.015 PMC2441863

[bib56] Barnett JH Scoriels L Munafo MR Meta-analysis of the cognitive effects of the catechol-O-methyltransferase gene Val158/108Met polymorphism Biol Psychiatry 2008 64 137 144 18339359 10.1016/j.biopsych.2008.01.005

[bib57] Munafo MR Clark TG Flint J Assessing publication bias in genetic association studies: evidence from a recent meta-analysis Psychiatry Res 2004 129 39 44 15572183 10.1016/j.psychres.2004.06.011

[bib58] Wardle MC de Wit H Penton-Voak I Lewis G Munafò MR Lack of association between COMT and working memory in a population-based cohort of healthy young adults Neuropsychopharmacology 2013 38 1253 1263 23337869 10.1038/npp.2013.24 PMC3656369

[bib59] Smoller JW Craddock N Kendler K Lee PH Neale BM Nurnberger JI Identification of risk loci with shared effects on five major psychiatric disorders: a genome-wide analysis Lancet 2013 381 1371 1379 23453885 10.1016/S0140-6736(12)62129-1 PMC3714010

[bib60] Schott BH Assmann A Schmierer P Soch J Erk S Garbusow M Epistatic interaction of genetic depression risk variants in the human subgenual cingulate cortex during memory encoding Transl Psychiatry 2014 4 e372 24643163 10.1038/tp.2014.10 PMC3966038

[bib61] Rajadhyaksha A Husson I Satpute SS Kuppenbender KD Ren JQ Guerriero RM L-type Ca2+ channels mediate adaptation of extracellular signal-regulated kinase 1/2 phosphorylation in the ventral tegmental area after chronic amphetamine treatment J Neurosci 2004 24 7464 7476 15329393 10.1523/JNEUROSCI.0612-04.2004 PMC1201527

[bib62] Dao DT Mahon PB Cai X Kovacsics CE Blackwell RA Arad M Mood disorder susceptibility gene CACNA1C modifies mood-related behaviors in mice and interacts with sex to influence behavior in mice and diagnosis in humans Biol Psychiatry 2010 68 801 810 20723887 10.1016/j.biopsych.2010.06.019 PMC2955812

[bib63] Licata SC Freeman AY Pierce-Bancroft AF Pierce RC Repeated stimulation of L-type calcium channels in the rat ventral tegmental area mimics the initiation of behavioral sensitization to cocaine Psychopharmacology 2000 152 110 118 11041323 10.1007/s002130000518

[bib64] Der-Avakian A D'Souza MS Pizzagalli DA Markou A Assessment of reward responsiveness in the response bias probabilistic reward task in rats: implications for cross-species translational research Transl Psychiatry 2013 3 e297 23982629 10.1038/tp.2013.74 PMC3756297

